# Amiodarone improves anemia in a murine model of sickle cell disease and is associated with increased erythrocyte bis(monoacylglycerol) phosphate

**DOI:** 10.1038/s41598-022-20955-5

**Published:** 2022-09-30

**Authors:** Jessica Venugopal, Jintao Wang, Chiao Guo, Daniel T. Eitzman

**Affiliations:** Cardiovascular Research Center, University of Michigan Internal Medicine-Cardiology Division, 7301A MSRB III, 1150 West Medical Center Drive, Ann Arbor, MI 48109-0644 USA

**Keywords:** Haematological diseases, Sickle cell disease

## Abstract

Sickle cell disease (SCD) is associated with altered plasma and erythrocyte lipid profiles. In a previous study, SCD mice with deficiency of proprotein convertase subtilisin/kexin type 9 (PCSK9) were observed to have more severe anemia and increased sickling compared to control SCD mice. Although PCSK9 affects circulating low density lipoprotein (LDL) by regulation of the LDL receptor, the effect of PCSK9 on anemia was independent of LDL receptor expression. In the current study, erythrocyte metabolomics were performed and revealed altered erythrocyte lipid species between SCD mice with and without PCSK9. Of particular interest, the late endosome-specific lipid bis(mono)acylglycerol phosphate (BMP) 44:12 was markedly decreased in erythrocytes from SCD mice deficient in PCSK9 mice relative to control SCD mice. Incubation of sickle erythrocytes with a neutralizing antibody to BMP increased erythrocyte sickling in vitro. In vitro treatment of SCD erythrocytes with amiodarone (1.5 μM) or medroxyprogesterone (6.75 μM), two pharmacologic compounds known to increase BMP, resulted in reduced erythrocyte sickling. Treatment of SCD mice with amiodarone (10 mg/kg) for 2 weeks resulted in increased BMP, improvement in anemia with reduced reticulocytosis, and decreased ex vivo sickling. In conclusion, severity of anemia in SCD is improved with amiodarone treatment, an effect which may be mediated through increased erythrocyte BMP.

## Introduction

Impaired erythropoiesis may play a role in the severity of sickle cell disease^1,2^. Preclinical studies with interventions shown to improve erythrocyte maturation by induction of autophagy or mitophagy have been shown to lessen SCD severity^3–5^. Autophagy and endosomal pathways intersect at several stages^6^ and endosomes are involved in erythrocyte maturation/physiology at several steps^7,8^.

Bis(monoacylglycero)phosphate (BMP), also called lysobisphosphatidic acid, is a regulatory lipid that plays a key role in endosomal integrity and function^9^. Although BMP may be altered in various disease states^10^, the causal relationship between BMP and disease pathophysiology remains unclear. Progesterone, which increases BMP^11,12^, may improve sickling phenotypes in humans. Another drug which increases BMP, Amiodarone^13^, has been effectively used in a SCD subject to treat ventricular arrythmias^11^.

Proprotein convertase subtilisin/kexin type 9 (PCSK9) is a protease produced primarily by the liver that targets the low density lipoprotein (LDL) receptor for degradation^14–16^. Deficiency of PCSK9 leads to lower levels of circulating LDL and protection from atherosclerotic cardiovascular disease^14–16^. Other LDLR-independent effects of PCSK9 have been described including effects on lysosomal degradation of other receptors related to the LDLR^17,18^. Although lipid lowering may be beneficial in SCD patients, SCD mice deficient in PCSK9 were shown to have more severe anemia with increased hemolysis^19^, effects that were found to be independent of LDLR expression^19^.

The objective of the current study was to pursue potential mechanisms related to the deleterious downstream effects of PCSK9 deficiency in SCD that might be evident in erythrocyte lipid species. Following a lipidomics screen using erythrocytes from SCD mice with PCSK9 deficiency, BMP was found to be markedly reduced in SCD mice with PCSK9 deficiency. In vitro and in vivo interventional studies were then performed to determine the potential biological significance of reduced erythrocyte BMP in SCD.

## Results and discussion

### BMP significantly decreased in PCSK9^-/-SCD^ erythrocytes

**Figure 1:**
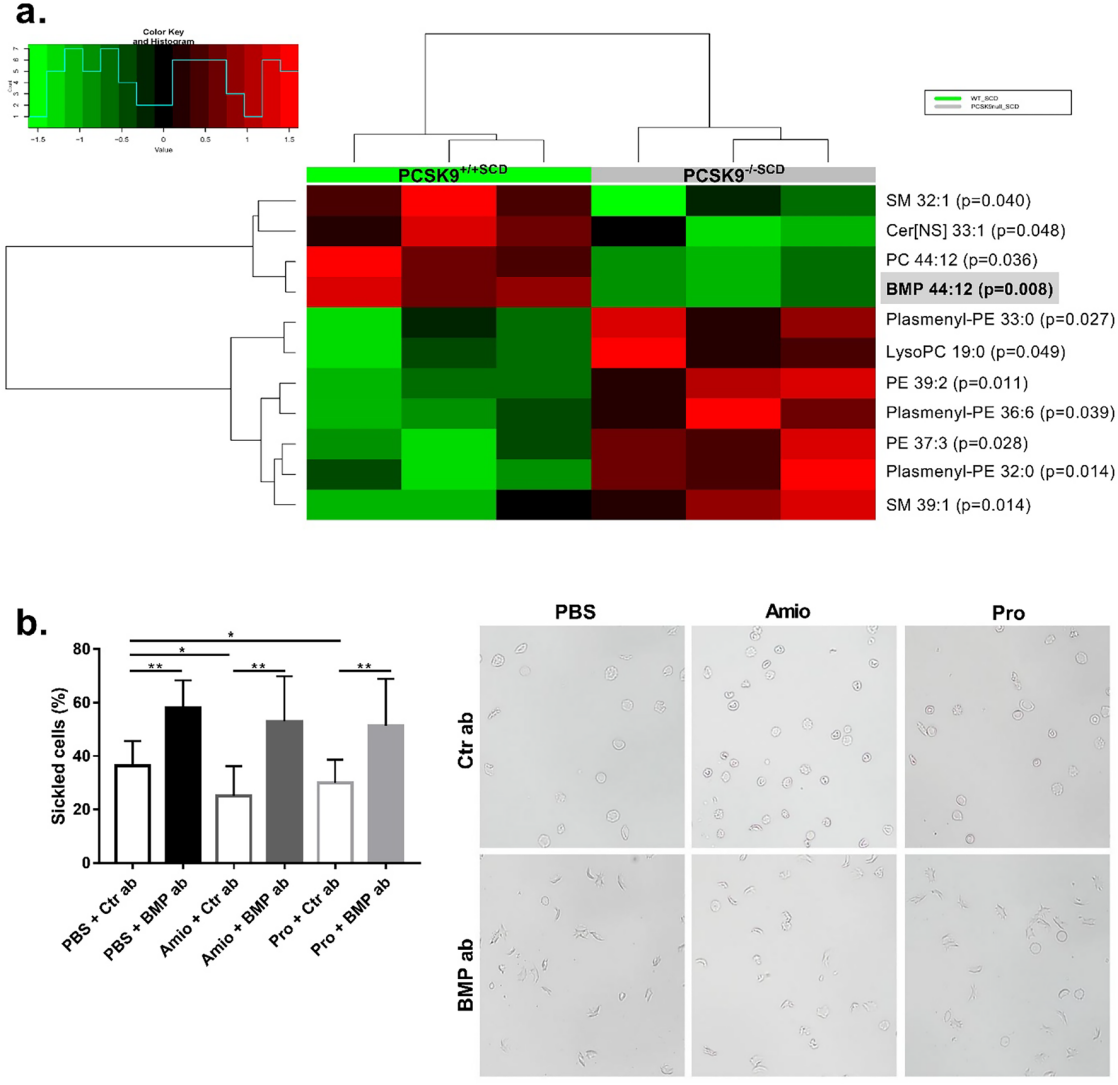
Decreased expression of BMP is associated with increased sickling. (**A**) Heat map of significantly altered lipid species between washed erythrocytes drawn from SCD male mice, either PCSK9 deficient or replete (n = 3 per group, 16 weeks post-BMT). The p value of each lipid is listed to the right of the heat map.* SM* sphingomyelin,* Cer* ceramide,* PC* phophocholine,* PE* phosphatidylethanolamine. (**B**) Quantification of the percentage of sickled erythrocytes ± standard deviation after incubation with anti-IgG, anti-BMP antibody, Amiodarone (Amio, 1.5 μM), and/or Medroxyprogesterone acetate (Pro, 6.75 μM) for 4 h, with representative images (n = 4 mice, 3 fields of view each mouse). Significance was determined by an independent one-way ANOVA, followed by a post-hoc analysis with Turkey’s multiple comparisons tests (*p < 0.05, **p < 0.01).

To investigate mechanism(s) underlying the more severe anemia and hemolysis observed in SCD mice with PCSK9 deficiency^19^, erythrocytes collected from control SCD mice and PCSK9 deficient SCD mice underwent untargeted lipidomics analysis to test the hypothesis that PCSK9 may affect erythrocyte lipids. Of 543 lipid species analyzed, 11 lipid species were different between the 2 groups of mice (Fig. 1A). Of particular interest, bismonoacylglycerol phosphate (BMP) 44:12 was markedly decreased in PCSK9^-/-SCD^ samples relative to PCSK9^+/+SCD^ samples (p = 0.008). BMP, also known as lysobisphosphotatidic acid, is a late endosomal lipid which is produced during maturation of the endosomes^20^. Endosomal function may play a critical role in reticulocyte maturation^21,22^.

### Antibody neutralization of BMP promotes sickling while drugs that increase BMP reduce sickling ex vivo

To determine a potential causal relationship between reduced erythrocyte BMP and in vitro sickling, washed SCD erythrocytes were incubated with a neutralizing anti-BMP antibody (50 μg/mL) for 4 h^23,24^, then the percentage of sickled cells were quantitated^25,26^. Erythrocytes incubated with the anti-BMP antibody showed increased sickling relative to erythrocytes treated with an anti-IgG control antibody (Fig. 1B), suggeting erythrocyte endosome trafficking during the sickling process may be regulated by BMP. The antiarrhythmic drug amiodarone has been shown to increase endosomal accumulation of BMP^27,28^, as has the steroid progesterone^29^. Both drugs have been detected in erythrocytes^30,31^. Decreased erythrocyte sickling occurred in response to both amiodarone and progesterone, and neither treatment was able to ameliorate the increased sickling in repsonse to the anti-BMP antibody (Fig. 1B). This suggests that these drugs reduce sickling by improving bioavailabiliy of BMP.

### Effect of amiodarone on sickling and anemia in SCD mice

**Figure 2:**
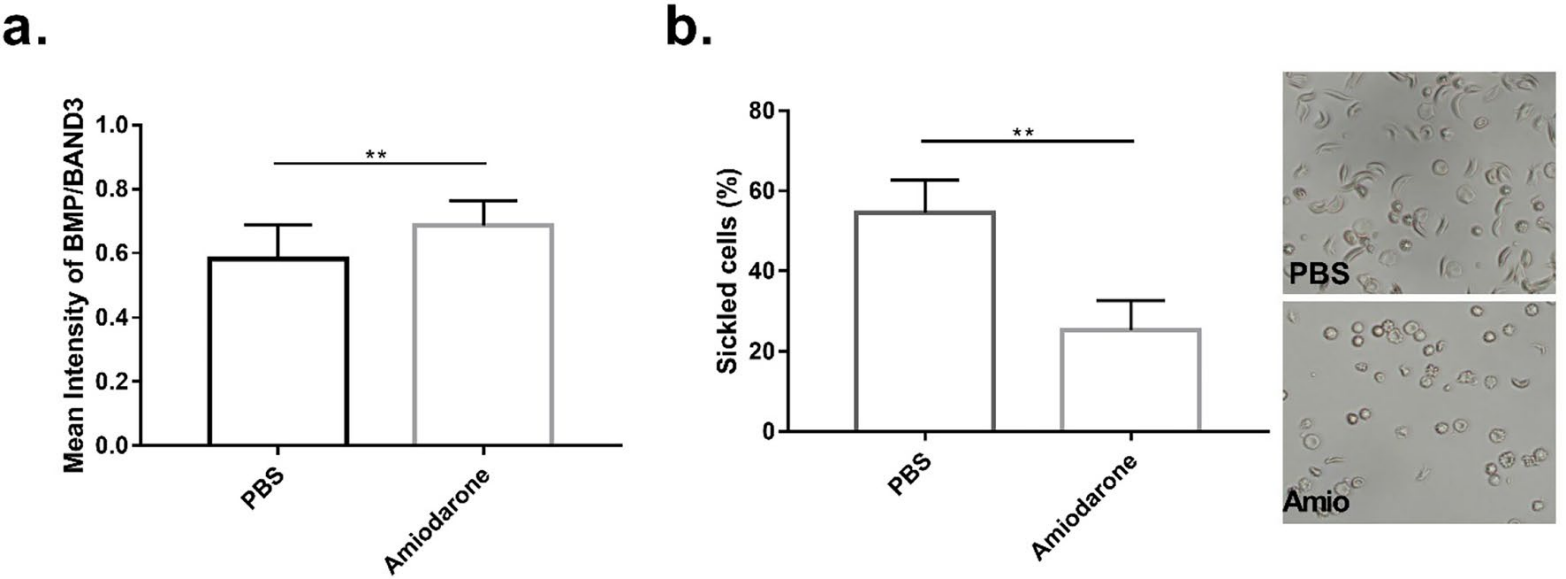
Amiodarone treatment of SCD mice increased erythrocyte BMP and decreased sickling. (**A**) Representative images of erythrocytes from mice treated with PBS or Amiodarone and incubated with anti-BMP and anti-BAND3 antibodies. Quantification of the mean signal intensity of anti-BMP reactivity divided by the mean signal intensity of anti-BAND3 reactivity of each cell (n = 4 mice each group, 3 fields of view each mouse). Error bars indicate standard deviation. (**B**) Quantification of the percentage of erythrocytes which had sickled after 2 h from PBS or Amiodarone treated mice ± standard deviation (n = 4 mice each group, 3 fields of view each mouse). Significance was determined by student’s t-test, (** = p < 0.01).

To determine the effect of amiodarone on erythrocyte BMP and anemia in SCD in vivo, SCD mice were given daily i.p. injections of amiodarone or vehicle for two weeks. BMP in erythrocytes from amiodarone-treated mice was increased compared to erythrocytes from vehicle-treated mice (Fig. 2A). This effect was associated with reduced ex vivo erythrocyte sickling (Fig. 2B). Furthermore, anemia was improved in mice treated with amiodarone as evidence by increased hemoglobin, hematocrit and erythrocytes (Table 1). Consistent with an effect on hemolysis, circulating reticulocytes were significantly decreased in mice treated with amiodarone (Table 1).

**Table 1:** Amiodarone treatment of SCD mice improved circulating RBC parameters.

Treatment (N)	RBC (M/uL)	HB (g/dL)	HCT (%)	Ret (%)	Sickle (%)
PBS (4)	7.88 ± 0.36	9.45 ± 0.48	38.00 ± 2.70	25.19 ± 3.87	54.54 ± 8.19
Amio (5)	8.08 ± 0.27	10.38 ± 0.43	41.25 ± 1.07	19.94 ± 1.17	25.46 ± 7.30
p value	0.046	0.0003	0.031	0.0366	0.012

Mice were given Amiodarone (10 mg/kg) or vehicle (PBS) via intraperitoneal injection daily for 2 weeks. Mean ± standard deviation listed, significance determined by student’s t-test (p < 0.05).

In conclusion, altered erythrocyte lipid profiles were identified in SCD mice with deficiency of PCSK9. Reduced erythrocyte BMP may contribute to more severe anemia and increased sickling tendency in PCSK9 deficient SCD mice. Consistently, pharmacologic targeting of erythrocyte BMP with amiodarone improves anemia in mice with SCD. Other pharmacologic strategies may be even more beneficial with reduced risk of side effects^29^. Several studies have found that sickle cell patients that use progestogen-only contraceptives have reduced pain crises^11,12^. Additional studies to determine downstream mechanisms for BMP effects on erythrocyte physiology are warranted. Although we suspect the effects of drug treatments in SCD are due to changes in erythrocyte lipids based on our previous work (Ref.^19^), further studies to characterize circulating lipid profiles in response to drug treatments with amiodarone and medroxyprogesterone acetate may be informative. Determination of in vivo effects of medroxyprogesterone acetate on anemia will also be necessary. For both treatments, effects on the downstream pathologic consequences of SCD related to release of free heme and consumption of nitric oxide may be revealing.

## Methods

### Animals

Male C57BL/6 J (Wildtype, *Wt*, stock # 000664), homozygous SCD mice (SCD, stock # 013071, Townes model) and PCSK9^-/-^ (stock # 005993) mice were purchased from Jackson Labs (Bar Harbor ME). Bone marrow was transplanted from SCD donor to PCSK9 deficient or WT recipient to generate the experimental PCSK9^-/-^SCD bmt mice and the PCSK9^+/+^SCD bmt controls, as previously^19^. All animal use protocols complied with the Principles of Laboratory and Animal Care established by the National Society for Medical Research and were approved by the University of Michigan Institutional Committee on Use and Care of Animals. The study is reported in accordance with ARRIVE guidelines.

### Lipidomics

Blood was drawn from the retro-orbital sinus of isoflourane-anesthetized mice (n = 3 per group) into 3.2% sodium citrate. Pelleted erythrocytes were washed 3 × with PBS and kept at – 80 ºC until analyzed by untargeted LC–MS Based Shotgun lipidomics by the Michigan Regional Comprehensive Metabolomics Resource Core. In short, lipids were extracted from samples using a modified Bligh-Dyer method^32^ analyzed by reversed-phase high performance liquid chromatography, followed by mass spectrometry analysis. Lipids were identified using the LipidBlast library^33^ and quantified using Multiquant, then normalized by internal standards.

### Amiodarone treatment

Amiodarone (10 mg/kg) (Hikma Farmaceutica, Portugal) or PBS (Gibco, pH 7.4) was injected daily (intraperitoneal, i.p.) into SCD mice for 2 weeks (n = 5 for Amiodarone and n = 4 for PBS).

### Complete blood counts

Blood samples were withdrawn from the retro-orbital venous plexus into EDTA-lined polythene tubes and were analyzed using a Hemavet 950 (Drew Scientific, Inc). An aliquot of whole blood was mixed with new methylene blue (Ricca Chemical Company) for 20 min to stain reticulocytes.

### Ex vivo sickling assays

For in vitro experiments, SCD mice (n = 4) were anesthetized using isofluorane, then blood was drawn from the retro-orbital venous plexus into EDTA-lined polythene tubes. SCD RBCs were washed 2 × with PBS then resuspended in PBS (30% HCT) and incubated with 50 μg/mL control IgG (Santa Cruz, # SC2025) or BMP antibody (6C4, END Millipore #MABT837) for 4 h at room temperature. Some erythrocytes were also incubated with 1.5 μM Amiodarone (Hikma Farmaceutica, Portugal) or 6.75 uM MedroxyPROGESTERone Acetate (Amphastar Pharmaceuticals Inc, USA) for 4 h. A 5 uL aliquot of blood was then placed on a slide with 5 μL sodium metabisulfite (Na_2_S_2_O_5_, Sigma, St. Louis, MO; 2% w/v in PBS), a cover slip applied, then 3 images per slide were captured at 20 × after a 60 min incubation at room temperature using a Nikon SE upright microscope and a Nikon DS-Fi3 camera. The percentage of sickled erythrocytes per image was quantified. Sodium metabisulfite is a reducing agent which scavenges oxygen, promoting deoxygenation in sickle cells^25,26^. For in vivo treatments, amiodarone- and vehicle-treated SCD mice were anesthetized using isoflurane, then blood was drawn from the retro-orbital venous plexus into EDTA-lined polythene tubes. Sickling percentages were determined in the same manner as in the whole blood sickling assays, described above.

### Fluorescence microscopy

Blood samples were withdrawn from the retro-orbital venous plexus into 3.2% sodium citrate, then centrifuged at 105×*g* for 5 min. The pellet was resuspended in PBS and then fixed for 20 min in 10% formalin + 0.2% glutaraldehyde. At the end of the incubation, cells were centrifuged at 1000 rpm for 5 min and then the pellet was resuspended in PBS. Fixed erythrocytes were permeabilized in 0.2% Triton + 3% BSA in PBS for 20 min. Cells were resuspended in PBS containing 3% BSA, 5 ug/mL anti-BMP antibody (6C4, END Millipore #MABT837), and 5 ug/mL anti-BAND3 antibody (Invitrogen, #PA5-80030), then incubated for 1 h at room temperature. Cells were washed 3 × with PBS then resuspended in anti-mouse FITC (Abcam, #ab6785; total conc = 2 mg/mL) and anti-rabbit Alexafluor-647 (LifeTechnologies, #A21446), then incubated for 1 h at room temperature while protected from light. After 3 washes, cell suspensions were placed on a glass slide containing Vectashield mounting medium (Vector Laboratories, #H-1400) and a glass coverslip was applied immediately. Three random fields of view at 20 × were imaged per sample and analysis was conducted with NIS-Elements (Nikon), using regions of interest to quantify the mean signal intensity for both BMP and BAND3 for each cell imaged.

### Statistical analysis

Data are represented as mean ± standard deviation. Statistical analysis was carried out using Graphpad Prism. Normality was determined with a Shapiro–Wilk Test. Significance was determined with a paired student’s t-test. For analysis of multiple groups, significance was determined by an independent one-way ANOVA, followed by a post-hoc analysis with Turkey’s multiple comparisons tests. Probability values of p < 0.05 were considered statistically significant.

## Data Availability

For original data, please contact deitzman@umich.edu.
